# Reviewed article: Penha SC, Carioca AAF, Pinto MS, Adriano LS, Bastos MF, Herculano RPB, Kendall BC, Kerr LRF S. Food and Nutrition Surveillance System: A time series analysis of data coverage, ultra-processed food consumption and obesity among pregnant adults and adolescents, Brazil, 2008-2022. Epidemiol Serv Saude. 2025:34;e20240713

**DOI:** 10.1590/S2237-96222025v34e20240713.a

**Published:** 2025-11-21

**Authors:** Samary Freire

**Affiliations:** 1 Universidade Federal do Rio de Janeiro, Rio de Janeiro, RJ, Brazil Rio de Janeiro RJ Brazil

## Peer review A

## Questionnaire

Does the manuscript contain new and significant information to justify publication? Yes

Does the Abstract (Summary) clearly and accurately describe the content of the article? Yes

Is the problem significant and concisely stated? Yes

Are the methods described comprehensively? No

Are the interpretations and conclusions justified by the results? Yes

Is adequate reference made to other work in the field? Yes

## Manuscript Structure

Length of article is: Adequate

Number of tables is: Adequate

Number of figures is: Adequate

Please state any conflict(s) of interest that you have in relation to the review of this paper. None

Interest: Good

Quality: Average

Originality: Average

Overall: Average

Recommendation: Minor Revision

## Comments

This study aims to analyze data’s trends of coverage from the Food and Nutrition Surveillance System and to reveal the prevalence of ultra-processed food consumption and obesity among Brazilian pregnant women between 2008 and 2022. This study uses secondary data available by SISVAN. The methodology needs to be a little more detailed to be reproducible and the discussion better written. The study reveals that there was an increase in obesity among adults and adolescents pregnant women, however the increase in the consumption of ultra-processed foods was not significant. Such findings prove to be relevant for the creation of public policies for this population. Some adjustments are needed.

